# Surgical Outcome After Distal Pancreatectomy With and Without Portomesenteric Venous Resection in Patients with Pancreatic Adenocarcinoma: A Transatlantic Evaluation of Patients in North America, Germany, Sweden, and The Netherlands (GAPASURG)

**DOI:** 10.1245/s10434-024-15932-3

**Published:** 2024-08-09

**Authors:** Thomas F. Stoop, Simone Augustinus, Bergthor Björnsson, Bobby Tingstedt, Bodil Andersson, Christopher L. Wolfgang, Jens Werner, Karin Johansen, Martijn W. J. Stommel, Matthew H. G. Katz, Michael Ghadimi, Michael G. House, Poya Ghorbani, I. Quintus Molenaar, Roeland F. de Wilde, J. Sven D. Mieog, Tobias Keck, Ulrich F. Wellner, Waldemar Uhl, Marc G. Besselink, Henry A. Pitt, Marco Del Chiaro, for the Global Audits on Pancreatic Surgery Group (GAPASURG), Tara M. Mackay, Salvador Rodriguez Franco, Susan Dierenvan Dieren

**Affiliations:** 1 Department of Surgery, Division of Surgical Oncology, University of Colorado Anschutz Medical Campus, Aurora, CO USA; 2 Amsterdam UMC, Location University of Amsterdam, Department of Surgery, Amsterdam, The Netherlands; 3 Cancer Center Amsterdam, Amsterdam, The Netherlands; 4 Division of Surgery, Department of Clinical Science, Intervention and Technology, Karolinska Institutet at Karolinska University Hospital, Stockholm, Sweden; 5 Department of Surgery in Linköping and Department of Biomedical and Clinical Sciences, Linköping University, Linköping, Sweden; 6 Department of Clinical Sciences Lund, Surgery, Skåne University Hospital, Lund University, Lund, Sweden; 7 Division of Surgical Oncology, Department of Surgery, New York University Medical Center, New York City, NY USA; 8 Department of General, Visceral and Transplant Surgery, University Hospital, LMU Munich, Munich, Germany; 9 Department of Surgery, Radboud University Medical Center, Nijmegen, The Netherlands; 10 Department of Surgical Oncology, The University of Texas MD Anderson Cancer Center, Houston, TX USA; 11 Department of General and Visceral Surgery, University Medical Center Göttingen, Göttingen, Germany; 12 Department of Surgery, Indiana University School of Medicine, Indianapolis, IN USA; 13 Department of Surgery, Regional Academic Cancer Center Utrecht, University Medical Center Utrecht/St. Antonius Ziekenhuis Nieuwegein, Utrecht & Nieuwegein, The Netherlands; 14 Department of Surgery, Erasmus MC Cancer Institute, University Medical Center, Rotterdam, The Netherlands; 15 Department of Surgery, Leiden University Medical Center, Leiden, The Netherlands; 16 DGAV StuDoQ|Pancreas and Clinic of Surgery, UKSH Campus Lübeck, Lübeck, Germany; 17 Department of Surgery, St. Josef Hospital, Ruhr University Bochum, Bochum, Germany; 18 Rutgers Cancer Institute of New Jersey, New Brunswick, NJ USA

**Keywords:** Distal pancreatectomy, Venous resection, Surgical outcome, Pancreatic cancer

## Abstract

**Background:**

Pancreatic adenocarcinoma located in the pancreatic body might require a portomesenteric venous resection (PVR), but data regarding surgical risks after distal pancreatectomy (DP) with PVR are sparse. Insight into additional surgical risks of DP-PVR could support preoperative counseling and intraoperative decision making. This study aimed to provide insight into the surgical outcome of DP-PVR, including its potential risk elevation over standard DP.

**Methods:**

We conducted a retrospective, multicenter study including all patients with pancreatic adenocarcinoma who underwent DP ± PVR (2018–2020), registered in four audits for pancreatic surgery from North America, Germany, Sweden, and The Netherlands. Patients who underwent concomitant arterial and/or multivisceral resection(s) were excluded. Predictors for in-hospital/30-day major morbidity and mortality were investigated by logistic regression, correcting for each audit.

**Results:**

Overall, 2924 patients after DP were included, of whom 241 patients (8.2%) underwent DP-PVR. Rates of major morbidity (24% vs. 18%; *p* = 0.024) and post-pancreatectomy hemorrhage grade B/C (10% vs. 3%; *p* = 0.041) were higher after DP-PVR compared with standard DP. Mortality after DP-PVR and standard DP did not differ significantly (2% vs. 1%; *p* = 0.542). Predictors for major morbidity were PVR (odds ratio [OR] 1.500, 95% confidence interval [CI] 1.086–2.071) and conversion from minimally invasive to open surgery (OR 1.420, 95% CI 1.032–1.970). Predictors for mortality were higher age (OR 1.087, 95% CI 1.045–1.132), chronic obstructive pulmonary disease (OR 4.167, 95% CI 1.852–9.374), and conversion from minimally invasive to open surgery (OR 2.919, 95% CI 1.197–7.118), whereas concomitant PVR was not associated with mortality.

**Conclusions:**

PVR during DP for pancreatic adenocarcinoma in the pancreatic body is associated with increased morbidity, but can be performed safely in terms of mortality.

Pancreatic adenocarcinoma may present with involvement of the portomesenteric vein.^1^ Nowadays, major venous tumor involvement is not considered a contraindication for surgical resection,^2,3^ particularly due to the increasing use of preoperative chemotherapy and improved surgical techniques.^4^ Since approximately two-thirds of the pancreatic adenocarcinomas are located in the pancreatic head,^5^ pancreatoduodenectomy (PD) with portomesenteric venous resection (PVR) is the most commonly performed procedure in patients with portomesenteric venous involvement. PD-PVR is associated with similar or slightly elevated surgical risks in comparison with standard PD without PVR.^6–8^ However, evidence on pancreatic adenocarcinoma located in the pancreatic body requiring a distal pancreatectomy (DP) with concomitant PVR is sparse and is thus far based only on single-center series.^9–12^

DP-PVR is a rarely performed procedure,^11,13^ which comprises 4–13% of all pancreatectomies where a concomitant PVR is needed.^14–20^ Performing DP-PVR is challenging due to restricted mobilization of the portomesenteric venous axis as the pancreatic head remains in situ. Depending on the tumor size, surgeons may consider an extended PD, or total pancreatectomy (TP) could be considered as alternatives to facilitate the venous reconstruction; however, TP is associated with endocrine and exocrine insufficiency,^21^ although these are more manageable nowadays with acceptably reduced quality of life.^22,23^ Moreover, PD is associated with higher risks for major morbidity and mortality in comparison with DP.^24,25^

More insight into the surgical outcome after DP-PVR in comparison with standard DP is needed for preoperative counseling and surgical decision making. Therefore, the present international observational study aimed to compare the surgical outcome after DP-PVR versus standard DP.

## Methods

This retrospective, observational, multicenter study was performed following the Strengthening the Reporting of Observational Studies (STROBE) guidelines.^26^ Informed consent was not necessary owing to the use of anonymous datasets.

### Study Design and Patients

All patients who were registered in the four registries of the Global Audits on Pancreatic Surgery Group (GAPASURG)^27^ between 2018 and 2020 who underwent DP for pancreatic adenocarcinoma were included, with or without PVR. Patients were included regardless of the surgical approach (i.e., minimally invasive or open), and regardless of upfront surgery or surgery following preoperative chemo(radio)therapy. Patients were excluded in the case of concomitant colon, gastric, and/or arterial resection(s),^28,29^ and/or a histopathological diagnosis other than pancreatic adenocarcinoma.

The GAPASURG database comprises the following four registries: (1) North America (American College of Surgeons National Surgical Quality Improvement Program [NSQIP], multicenter, 160 centers in 2019), Germany (Deutsche Gesellschaft für Allgemein- und Viszeralchirurgie—Studien-, Dokumentations-und Qualitätszentrum [StuDoQ]^30^, multicenter, 58 centers in 2019), Sweden (Swedish National Pancreatic and Periampullary Cancer Registry,^31^ nationwide, 6 centers in 2020), and The Netherlands (Dutch Pancreatic Cancer Audit,^32^ nationwide, 17 centers in 2019). Differences in registry design among these four registries are presented in “Appendix 1”.

### Endpoints

The primary endpoints were in-hospital/30-day mortality and major morbidity, while secondary endpoints concerned pancreas surgery-specific complications (i.e., post-pancreatectomy hemorrhage [PPH], postoperative pancreatic fistula [POPF], delayed gastric emptying [DGE]), relaparotomy rate, length of hospital stay, and rate of readmission.

### Definitions

Differences in parameters between registries due to the different metric systems were resolved by converting the data; ounces were converted to kilograms and inches into meters. Several variables were recategorized, such that data could be combined.

The Eastern Cooperative Oncology Group (ECOG) performance status was recategorized to match functional health status of being independent (ECOG 0–1), partially dependent (ECOG 2–3), and totally dependent (ECOG 4). In addition, the American Society of Anesthesiologists performance status (ASA-PS) is used.

Hospital volumes for pancreatic surgery are classified based on the annual number of PDs performed; dichotomized into low-volume (i.e., < 40 PDs/year) and high-volume (i.e., ≥ 40 PDs/year) hospitals. The annual PD volume was used as this better illustrates the experience with more complex pancreatic surgery compared with the annual DP volume.^33^ Not all registries contained information on the type of PVR resection and reconstruction;^3^ therefore, no distinction is made between the PVR type. Furthermore, portomesenteric venous tangential resections with primary closure (International Study Group of Pancreatic Surgery [ISGPS] type 1)^3^ are not included in the NSQIP as PVR.

All complications and mortality were measured during a 30-day postoperative follow-up. In addition, in-hospital events, when hospital stay exceeded > 30 days, were registered, except within the NSQIP, in which only 30-day follow-up was registered. Major morbidity was defined as Clavien–Dindo grade IIIa or higher.^34^ Pancreas surgery-specific complications (i.e., POPF, DGE, and PPH) were defined in accordance with the ISGPS definitions, whereby grade B/C is considered as clinically relevant.^35–37^ Readmission was defined as any readmission (i.e., to the same or another hospital) for any reason within 30 days after the principal operative procedure.

Within North America, Germany, and The Netherlands, the American Joint Committee on Cancer (AJCC) TNM 8th edition was used to determine the T and N stage, whereas Sweden used the AJCC 7th edition. For the current study, the (y)pTNM parameters are categorized into T0–T3 versus T4, and N0 versus N1–2. Resection margin status (R status) was defined following the Royal College of Pathologists definition.^38^

### Statistical Analyses

Statistical analyses were performed using IBM SPSS Statistics for Windows (version 28.0.1.1; IBM Corporation, Armonk, NY, USA). Statistical significance was reached in case of a two-tailed *p* value < 0.050.

Categorical variables are presented as percentages and frequencies, analyzed using Pearson’s Chi square test or Fisher’s exact test where appropriate. Continuous variables with normal distributions are presented as means with standard deviations (± SD) and compared with the independent Student *t* test. Non-normally distributed continuous variables are presented as medians with interquartile ranges (IQRs) and compared using the Mann–Whitney *U* test.

A missing data category was introduced for each categorical variable with ≥ 2% missing data. Otherwise, patients with missing data for variables missing < 2% were excluded (arbitrary cut-off). If a categorical variable had < 2% missing data, the missing patients were not included in the overall proportions reported, and they were not included in the test of association. If a categorical variable had ≥ 2% missing, these patients were classified as missing and were included in the overall proportions and the test of association. For all continuous variables, the missing patients were not included in the test of association. This strategy was used for both the descriptive analyses and the logistic regression analyses.

Some variables of interest were not collected in all four registries during the study period (i.e., hospital volume, intraoperative blood loss, PPH, admission to the intensive care unit [ICU], and R status were not collected in the NSQIP), and were therefore categorized as ‘not available’; this category was not tested in the test of association, except for the logistic regression analyses when applicable.

Logistic regression analyses were performed to investigate the potential predictors for major morbidity and mortality, whereby the results are presented in odds ratios (ORs) with 95% confidence intervals (CIs). Independent variables with *p* < 0.050 in the univariable analysis were subsequently tested in the multivariable analysis. When an independent variable was significant based on only the ‘missing data’ category, this variable was not used in the selection either for or within the multivariable analysis. In the multivariable analysis, backward selection was used until the model only contained independent parameters that were statistically significant.

## Results

A total of 9801 DPs were performed during the study period, of which 6877 patients (70.2%) were excluded because of a diagnosis other than pancreatic adenocarcinoma (*n* = 6177, 63.0%), had missing data regarding the diagnosis (*n* = 260, 2.7%), had missing data on whether a vascular resection was performed (*n* = 45, 0.5%), or underwent DP with concomitant arterial resection[s] (*n* = 168, 1.7%) or multivisceral resection[s] (*n* = 227, 2.3%).

The final study cohort comprised 2924 patients, of whom 8.2% (*n* = 241) underwent a concomitant PVR. In North America, Germany, Sweden, and The Netherlands, the proportion of patients who underwent a concomitant PVR among the included patients was 9.2% (*n* = 199/2165), 5.9% (*n* = 27/461), 1.9% (*n* = 3/157), and 8.5% (*n* = 12/141), respectively (*p* = 0.002).

### Baseline Characteristics

See Table 1 for the baseline characteristics. Patients undergoing DP-PVR were younger (mean 67 ± 10 years) in comparison with patients who underwent a standard DP (mean 69 ± 10; *p* = 0.004), were more likely to be female (*n* = 1342 [50.0%] vs.* n* = 143 [59.3%]; *p* < 0.006), and had a lower mean body mass index (27 ± 6 vs. 26 ± 6; *p* = 0.040). The performance status and the prevalence of cardiovascular and pulmonary comorbidities were similar between both groups. More patients undergoing DP-PVR were treated with preoperative chemotherapy than patients without PVR (*n* = 124 [51.5%] vs.* n* = 702 [26.2%]; *p* < 0.001).

**Table 1:** Baseline characteristics

Variables	Standard DP (*n* = 2683)	DP + PVR (*n* = 241)	*p* value
Registry			**0.002**^a^
North America	1966 (73.3)	199 (82.6)	
Germany	434 (16.2)	27 (11.2)	
Sweden	154 (5.7)	3 (1.2)	
The Netherlands	129 (4.8)	12 (5.0)	
Age, years (mean ± SD)	69 ± 10	67 ± 10	**0.004**^b^
Female	1342 (50.0)	143 (59.3)	**0.006**^a^
BMI, kg/m^2^ (mean ± SD)	27 ± 6	26 ± 6	**0.040**^b^
Missing (*n*)	14	2	
ASA-PS			0.101^a^
I–II	767 (28.6)	57 (23.7)	
III–IV	1913 (71.4)	184 (76.3)	
Missing	3	0	
Performance status			0.452^a^
Independent	2589 (97.6)	235 (98.3)	
Dependent^c^	65 (2.4)	4 (1.7)	
Missing	29	2	
Congestive heart failure	98 (3.7)	7 (2.9)	0.540^a^
Missing	15	0	
COPD	162 (6.0)	15 (6.2)	0.913^a^
Missing	5	0	
Diabetes mellitus	829 (31.0)	73 (30.3)	0.830^a^
Missing	5	0	
Preoperative chemotherapy	702 (26.2)	124 (51.5)	**< 0.001**^a^
Missing	60 (2.2)	4 (1.7)	

Bold values indicate statistical significance (*p* < 0.050)

Data are expressed as *n* (%) unless otherwise specified

If the ‘missing’ percentage is reported, it is included in the test of association. Otherwise, patients with missing data were removed for the test of association

*n* number of patients, *DP* distal pancreatectomy, *PVR* portomesenteric venous resection, *SD* standard deviation, *BMI* body mass index, *ASA-PS* American Society of Anesthesiologists performance status, *COPD* chronic obstructive pulmonary disease

^a^Chi-square test

^b^Student’s *t* test

^c^*n* = 1 patient was classified as being fully dependent, whereas all other patients were partially dependent.

### Surgery Details and Surgical Outcome

Details regarding surgery and surgical outcomes are presented in Table 2. The rate of primary open surgery was higher in the DP-PVR group (*n* = 199, 82.6%) compared with the standard DP group (*n* = 1626, 60.7%) [*p* < 0.001]. Among the patients who underwent minimally invasive surgery, the conversion rate was higher in the DP-PVR group (*n* = 19/36, 52.8%) compared with the standard DP group (*n* = 212/875, 24.2%) [*p* < 0.001].

**Table 2:** Surgery and surgical outcome

Variables	Standard DP (*n* = 2683)	DP + PVR (*n* = 241)	*p* value
Annual PD volume^d^			0.931^a^
Low	342 (48.3)	20 (47.6)	
High	366 (51.7)	22 (52.4)	
Missing	9	2	
Not available	1966	199	
Surgical approach			**< 0.001**^a^
Open	1626 (60.7)	199 (82.6)	
Minimally invasive	663 (24.7)	17 (7.1)	
Minimally invasive with conversion	212 (7.9)	19 (7.9)	
Others	178 (6.6)	6 (2.5)	
Missing	4	0	
Operation time, min [median (IQR)]	223 (167–295)	318 (221–405)	**< 0.001**^b^
Missing (*n*)	480	32	
Intraoperative blood loss, mL [median (IQR)]^e^	200 (100–480)	400 (225–1115)	**0.043**^b^
Missing (*n*)	478	29	
Not available (*n*)	1966	199	
Delayed gastric emptying			0.227^a^
None or grade A	2550 (95.5)	225 (93.8)	
Grade B/C	121 (4.5)	15 (6.3)	
Missing	12	1	
Postpancreatectomy hemorrhage^d^			**0.041**^c^
None or grade A	696 (97.1)	37 (90.2)	
Grade B/C	21 (2.9)	4 (9.8)	
Missing	0	1	
Not available	1966	199	
Postoperative pancreatic fistula			0.466^a^
None	2333 (87.7)	204 (86.1)	
Grade B/C	327 (12.3)	33 (13.9)	
Missing	23	4	
Major morbidity	479 (17.9)	57 (23.8)	**0.024**^**a**^
Missing	4	1	
Relaparotomy	94 (3.5)	12 (5.0)	0.237^a^
Missing	4	1	
ICU admission^d^	24 (3.4)	4 (9.8)	0.060^c^
Missing	7	1	
Not available	1966	199	
Mortality	33 (1.2)	4 (1.7)	0.542^c^
Missing	1	4	
Length of hospital stay, days [median (IQR)]	6 (5–10)	7 (6–11)	**< 0.001**^**b**^
Missing (*n*)	22	0	
Readmission	386 (14.4)	36 (15.0)	0.803^a^
Missing	4	1	

Bold values indicate statistical significance (*p* < 0.050)

Data are expressed as *n* (%) unless otherwise specified

If the ‘missing’ percentage is reported, it is included in the test of association. Otherwise, patients with missing data were removed for the test of association

*DP* distal pancreatectomy, *PVR* portomesenteric venous resection, *n* number of patients, *PD* pancreatoduodenectomy, *IQR* interquartile range, *ICU* intensive care unit, *NSQIP* National Surgical Quality Improvement Program

^a^Chi-square test

^b^Mann–Whitney U test

^c^Fisher’s exact test

^d^Data are not available in the NSQIP

^e^Data are not available in the NSQIP and StuDoQ

The rate of major morbidity was higher after DP-PVR versus standard DP (*n* = 57 [23.8%] vs.* n* = 479 [17.9%]; *p* = 0.024), without significant differences in the relaparotomy rate (*n* = 12 [5.0%] vs.* n* = 94 [3.5%]; *p* = 0.237) and ICU admission (*n* = 4 [9.8%] vs.* n* = 24 [3.4%]; *p* = 0.060). However, the rate of PPH grade B/C was higher after DP-PVR (*n* = 4, 9.8%) in comparison with standard DP (*n* = 21, 2.9%) [*p* = 0.041]. From those patients who developed PPH grade B/C, 75.0% (*n* = 3/4) of patients after DP-PVR and 47.6% (*n* = 10/21) after standard DP also had POPF grade B/C (*p* = 0.593). In the subgroup of patients who developed POPF grade B/C (with available data on PPH), the rate of PPH grade B/C was higher after DP-PVR compared with standard DP (*n* = 3/10 [30.0%] vs.* n* = 10/133 [7.5%]; *p* = 0.049). The median length of hospital stay was longer after DP-PVR (7 days [IQR 6–11]) compared with patients who underwent standard DP (6 days [IQR 5–10]) [*p* < 0.001].

The in-hospital/30-day mortality did not differ between DP-PVR and standard DP (*n* = 4 [1.7%] vs.* n* = 33 [1.2%]; *p* = 0.542). Mortality rates among patients undergoing primary open DP-PVR versus minimally invasive DP-PVR versus minimally invasive surgery requiring conversion to open were 2.0% (*n* = 4/199), 0% (*n* = 0/17), and 0% (*n* = 0/19) [*p* > 0.999], respectively. See Table 3 for the histopathological outcome.

**Table 3:** Histopathological outcome

Variables	Standard DP (*n* = 2683)	DP + PVR (*n* = 241)	*p* value
(y)pT stage			**< 0.001**^a^
T0–T3	2554 (95.2)	223 (92.5)	
T4	45 (1.7)	14 (5.8)	
Tx	7 (0.3)	1 (0.4)	
Missing	77 (2.9)	3 (1.2)	
(y)pN stage			0.136^b^
N0	1319 (49.2)	111 (46.1)	
N1–2	1240 (46.2)	124 (51.5)	
Missing	124 (4.6)	6 (2.5)	
Residual disease^c^			**< 0.001**^b^
R0	536 (74.8)	18 (42.9)	
R1	157 (21.9)	22 (52.4)	
Missing	24 (3.3)	2 (4.8)	
Not available	1966	199	

Bold values indicate statistical significance (*p* < 0.050)

Data are expressed as *n* (%)

If the ‘missing’ percentage is reported, it is included in the test of association. Otherwise, patients with missing data were removed for the test of association

*n* number of patients, *DP* distal pancreatectomy, *PVR* portomesenteric venous resection, *NSQIP* National Surgical Quality Improvement Program

^a^Fisher’s exact test

^b^Chi-square test

^c^Data are not available in the NSQIP

### Predictors for Major Morbidity and Mortality

See Table 4 for the logistic regression analysis for predictors of major morbidity. PVR was an independent predictor for major morbidity (OR 1.500, 95% CI 1.086–2.071). Minimally invasive surgery converted to open surgery was also associated with major morbidity (OR 1.420, 95% CI 1.023–1.970). On the other hand, preoperative chemotherapy was associated with a lower likelihood of developing major morbidity (OR 0.765, 95% CI 0.611–0.959). Moreover, the Swedish registry was associated with less major morbidity.

**Table 4:** Predictors for major morbidity

Variables	*n*	Univariable analysis			Multivariable analysis		
		OR	95% CI	*p* value	OR	95% CI	*p* value
Registry							
North America	2165		1 (Referent)			1 (Referent)	
Germany	461	1.171	0.912–1.504	0.215	1.130	0.870–1.467	0.361
Sweden	156	0.509	0.300–0.863	**0.012**	0.503	0.295–0.858	**0.012**
The Netherlands	137	1.093	0.708–1.688	0.688	1.115	0.664–1.871	0.682
Age, years	2919	1.000	0.991–1.009	0.963	–	–	–
BMI, kg/m^2^							
Normal weight (18.5–25.0)	1106		1 (Referent)		–	–	–
Underweight (< 8.5)	67	0.923	0.475–1.795	0.814			
Overweight (25.0–30.0)	1009	1.069	0.857–1.335	0.553			
Obesity (≥ 30.0)	721	1.133	0.890–1.441	0.311			
Performance status							
Independent	2819		1 (Referent)		–	–	–
Dependent	69	1.360	0.771–2.399	0.288			
Diabetes mellitus							
No	2012		1 (Referent)		–	–	–
Yes	902	1.128	0.923–1.378	0.238			
COPD							
No	2739		1 (Referent)		–	–	–
Yes	175	1.343	0.932–1.937	0.114			
Congestive heart failure							
No	2799		1 (Referent)		–	–	–
Yes	105	0.732	0.420–1.275	0.271			
Preoperative chemotherapy							
No	2032		1 (Referent)			1 (Referent)	
Yes	826	0.803	0.647–0.996	**0.046**	0.765	0.611–0.959	**0.020**
Missing	61	0.920	0.475–1.784	0.806	0.860	0.395–1.869	0.703
Annual PD volume							
Low	358		1 (Referent)		–	–	–
High	387	0.877	0.606–1.269	0.487			
Not available	2165	0.696	0.696–1.226	0.583			
Surgical approach							
Open	1820		1 (Referent)			1 (Referent)	
Minimally invasive	680	0.907	0.717–1.146	0.412	0.931	0.731–1.185	0.590
Minimally invasive with conversion	231	1.456	1.053–2.012	**0.023**	1.420	1.023–1.970	**0.036**
Other	184	1.145	0.783–1.674	0.485	1.149	0.779–1.695	0.485
Portomesenteric venous resection							
No	2679		1 (Referent)			1 (Referent)	
Yes	240	1.431	1.046–1.957	**0.025**	1.500	1.086–2.071	**0.014**

Bold values indicate statistical significance (*p* < 0.050)

*n* number of patients, *OR* odds ratio, *CI* confidence interval, *BMI* body mass index, *PD* pancreatoduodenectomy, *COPD* chronic obstructive pulmonary disease

See Table 5 for the logistic regression analysis for predictors of 30-day/in-hospital mortality. PVR was not associated with mortality (OR 1.355, 95% CI 0.476–3.857). A higher age (OR 1.087, 95% CI 1.045–1.132), chronic obstructive pulmonary disease (OR 4.167, 95% CI 1.852–9.374), and minimally invasive surgery converted to open surgery (OR 2.919, 95% CI 1.197–7.118) were independent predictors for mortality. The different registries were not associated with mortality.

**Table 5:** Predictors for mortality

Variables	*n*	Univariable analysis			Multivariable analysis		
		OR	95% CI	*p* value	OR	95% CI	*p* value
Registry							
North America	2165		1 (Referent)		–	–	–
Germany	461	1.268	0.547–2.940	0.579			
Sweden	156	0.531	0.072–3.937	0.536			
The Netherlands	141	1.788	0.535–5.982	0.345			
Age, years	2924	1.089	1.048–1.131	**< 0.001**	1.087	1.045–1.132	**< 0.001**
BMI, kg/m^2^							
Normal weight (18.5–25.0)	1106		1 (Referent)		–	–	–
Underweight (< 18.5)	68	3.017	0.655–13.889	0.156			
Overweight (25.0–30.0)	1012	1.094	0.472–2.534	0.834			
Obesity (≥ 30.0)	721	1.685	0.739–3.839	0.214			
Performance status							
Independent	2823		1 (Referent)		–	–	–
Dependent	69	1.171	0.158–8.675	0.877			
Diabetes mellitus							
No	2016		1 (Referent)		–	–	–
Yes	902	1.716	0.891–3.303	0.106			
COPD							
No	2741		1 (Referent)			1 (Referent)	
Yes	177	4.427	1.993–9.832	**< 0.001**	4.167	1.852–9.374	**< 0.001**
Congestive heart failure							
No	2803		1 (Referent)		–	–	–
Yes	105	NE	NE	–			
Preoperative chemotherapy							
No	2033		1 (Referent)		–	–	–
Yes	826	1.084	0.531–2.213	0.825			
Annual PD volume							
Low	362		1 (Referent)		–	–	–
High	387	0.777	0.235–2.567	0.679			
Not available	2165	0.721	0.295–1.765	0.474			
Surgical approach							
Laparotomy	1824		1 (Referent)			1 (Referent)	
Minimally invasive	680	1.562	0.739–3.300	0.242	1.496	0.703–3.180	0.296
Minimally invasive with conversion	231	2.969	1.234–7.140	**0.015**	2.919	1.197–7.118	**0.018**
Other	184	NE	NE	–	NE	NE	–
Portomesenteric venous resection							
No	2682		1 (Referent)		–	–	–
Yes	241	1.355	0.476–3.857	0.569			

Bold values indicate statistical significance (*p* < 0.050)

*n* number of patients, *OR* odds ratio, *CI* confidence interval, *BMI* body mass index, *PD* pancreatoduodenectomy, *NE* not estimable, *COPD* chronic obstructive pulmonary disease

## Discussion

To date, this largest observational international study in 2924 patients with pancreatic adenocarcinoma demonstrated that PVR during DP is associated with higher rates of in-hospital/30-day major morbidity (24% vs. 18%) and PPH grade B/C (10% vs. 3%), whereas it did not increase the in-hospital mortality/30-day major morbidity (1.7% vs. 1.2%). PVR was an independent predictor for developing major morbidity, but did not impact the mortality. In contrast, older age, chronic obstructive pulmonary disease, and conversion from minimally invasive to open surgery were associated with mortality. Nevertheless, no mortality occurred among patients who underwent DP-PVR after conversion from minimally invasive to open surgery.

Previously, two single-center, observational studies investigated the surgical outcome after DP-PVR versus standard DP.^9,11^ Loos et al. compared 79 patients after DP-PVR with 1383 patients who underwent standard DP for any indication, without concomitant arterial and/or multivisceral resection(s).^11^ Of note, the DP-PVR group was defined as either a concomitant PVR or left renal vein resection. Their univariable logistic regression analyses supported the findings from the current study that DP-PVR is associated with major morbidity but not with short-term mortality.^11^ However, no distinction was made between standard DP and DP-PVR in the multivariable analyses. Although Loos et al. did not perform direct statistical comparisons from DP-PVR versus standard DP, differences in surgical outcome between both procedures in that study seemed in line with our study: major morbidity (41% vs. 22%), PPH grade C (5% vs. 2%), and 90-day mortality (1% vs. 1%).^11^ A second single-center, observational study compared DP-PVR (*n* = 41) with standard DP (*n* = 82) without concomitant arterial resection(s), again performed for any indication.^9^ Both 90-day mortality (0% vs. 3%) and major morbidity (17% vs. 13%) did not differ significantly. However, the rate of PPH was significantly higher after DP-PVR (17% vs. 4%), as was also found in our study.^9^

Among patients who developed POPF grade B/C in this study, the rate of PPH grade B/C was higher after DP-PVR compared with standard DP (30% vs. 8%), but no information was available on the timing and cause of PPH. Unfortunately, only two of four registries/audits collected data on both PPH and intraoperative drain management, leaving too few patients to sufficiently investigate patterns in drain management between DP-PVR and standard DP. No difference in the rate of PPH grade B/C was observed in the international PANDORINA trial wherein 282 patients who underwent a DP were randomized between drain versus no drain placement.^39^ Since the detection of POPF grade B/C was lower in the no drain group, drain placement during DP-PVR might detect POPF in an earlier stage, hypothetically reducing the risk for PPH grade B/C from the reconstructed vein.^40^ Another potential cause of PPH is the use (and type) of thromboprophylaxis,^41,42^ but no data on the use of anti-coagulance were available—an important understudied topic with variances in local protocols.^42^

The findings from both single-center series^9,11^ suggest that, in particular, the increased risk for PPH after DP-PVR contributes to the higher major morbidity. The lack of significance in the current study from the differences in ICU admission and relaparotomy rates suggests that the complications related to PVR can mostly be managed with minimally invasive interventions (i.e., Clavien–Dindo grade IIIa^34^). In contrast, the results from the Heidelberg series suggested that the rates of relaparotomy and ICU admission are higher after PD-PVR compared with standard DP, although this observation could also be related to the higher rates of POPF after DP-PVR compared with standard DP (33% vs. 20%).^11^

The technical challenge of a DP-PVR is the limited mobilization of the portomesenteric venous axis, in contrast to a PD or TP due to removal of the pancreatic head and the Kocher and Cattell–Braasch maneuvers.^43^ As a consequence, a DP-PVR for a pancreatic body tumor is mostly only performed in the presence of limited left-sided venous involvement. Segmental venous resections are performed in 22–56% of the DP-PVRs.^9,14,18,19^ Hypothetically, the limited capability to mobilize the portomesenteric veins increases the risk for narrowing of the venous reconstruction and thus the risk for early portomesenteric venous thrombosis.^12^ Other known risk factors for thrombosis are the use of synthetic grafts for the venous reconstruction and performing a segmental venous resection.^14,44,45^ Three studies reported on the incidence of thrombosis after DP-PVR.^12,17,46^ A single-center experience from Roch et al. revealed that 4 of 14 patients (29%) developed portomesenteric venous thrombosis after DP-PVR for any indication.^17^ Another single-center study reported a 7% incidence of portomesenteric venous thrombosis among 15 patients who underwent DP-PVR for a locally advanced pancreatic neuroendocrine tumor,^46^ while a third single-center study described high rates of occlusion (22%) and > 50% narrowing (28%) among 32 patients who underwent a DP-PVR for pancreatic adenocarcinoma.^12^ Based on these findings, Maekawa et al. proposed to perform a PD-PVR instead of DP-PVR if the length of venous involvement is ≥ 30 mm.^12^ Unfortunately, information regarding the incidence of thrombosis was not available in the four registries used for the present study.

The results from the current study suggest that DP-PVR is associated with a significant but limited increased risk for major morbidity without an increase in short-term mortality in comparison with a standard DP. Therefore, DP-PVR seems to be a reasonably safe and feasible procedure in patients with pancreatic adenocarcinoma located in the pancreatic body, with limited portomesenteric venous involvement when performed by experienced surgeons. Importantly, the seemingly limited additional risks of DP-PVR over standard DP could be related to more high-volume surgeons/centers performing these procedures. However, this remains speculative because volume data are not available within the NSQIP. Nevertheless, this limited risk elevation seems to outbalance the risks and long-term sequalae from alternative procedures (i.e., extended PD-PVR or TP-PVR). Established benchmarks for major morbidity (≤ 28%) and in-hospital mortality (≤ 4%) after PD-PVR performed in a high-volume center are higher than the outcomes after DP-PVR in our study.^6^ Another option is a TP-PVR, which is associated with similar surgical outcomes as the benchmarks for PD-PVR when performed in high-volume centers.^47,48^ However, TP should be avoided when possible considering the associated endocrine and exocrine insufficiencies.^22^ Furthermore, TP-PVR is associated with gastric venous congestion, particularly when combined with left gastric vein ligation.^49^ Of note, it might also be needed to sacrifice the left gastric vein as part of DP-PVR, after which the gastric venous drainage depends on the right gastric vein, right gastroepiploic vein, and distal esophageal veins, which might be associated with gastric venous congestion. Eventually, the left gastric vein could be reconstructed.^50^ Since the extent of tumor involvement with the portomesenteric venous axis was not available in the currently used registries, it is possible that an extended PD or TP is a more safe and feasible procedure in the case of more extensive venous involvement, depending on the surgeon’s preference.

Over the last decade, the use of minimally invasive DP for benign and (pre-)malignant pancreatic diseases has increased.^51^ It is likely this trend will continue based on the results from the DIPLOMA trial, which demonstrated the non-inferiority of minimally invasive DP over open DP in patients with resectable pancreatic adenocarcinoma.^52^ The present observational study showed that conversion from minimally invasive to open surgery is independently associated with both in-hospital/30-day major morbidity and mortality. As vascular involvement is known to be the main indication to convert, caution is required for adenocarcinomas located in the pancreatic body with proximity to the portomesenteric venous axis.^53^ Unfortunately, the reasons for conversion to open surgery (e.g., unexpected vascular involvement, intraoperative bleeding) are not registered in the four audits/registries. It would seem prudent to use recent imaging when embarking on a minimally invasive DP with potential vascular involvement. The DIPLOMA trial required computed tomography at a maximum of 4 weeks before surgery.^48^

The present study showed that preoperative chemo(radio)therapy was associated with lower odds of developing major morbidity. This might be related to a lower rate of POPF as a consequence of radiotherapy, however data regarding treatment with preoperative radiotherapy were not available in all four registries.^54^

The results from this study should be seen in the light of several limitations. First, no data were available regarding either the extent of venous involvement or the type of PVR, which was why no proper comparison could be made with the GAPASURG data to compare DP-PVR with either TP-PVR or PD-PVR. Both PD and TP procedures can be performed for a wide spectrum of indications and with various concomitant procedures, associated with different levels of complexity and outcomes.^47,48,55^ Second, only ISPGS type 2–4 resections were registered as PVR in the NSQIP registry, which might have influenced the surgical outcome in this study. Third, no data were available regarding anti-coagulance therapy, short- and long-term patency of the venous reconstruction, timing and origin of PPH, and detailed data on perioperative oncological treatments and survival. Fourth, the rule of thumb for the logistic regression analysis on predictors for mortality was more or less violated, testing one more dependent variable than allowed, considering the number of events. Fifth, even though all patients who underwent an arterial resection were excluded, a minority of patients was classified as having (y)pT4, most likely due to a misclassification by the pathologist. Sixth, the small numbers of patients who underwent DP-PVR in Germany, The Netherlands, and Sweden did not allow comparative analyses between DP-PVR and standard DP for each of the four registries separately; therefore, the results could have been influenced by (potential) differences in clinical practices among countries (e.g., regarding patient selection, length of hospital stay) and the structure from the registries. Important differences between the registries were (1) voluntary and multicenter registries (North America and Germany) versus mandatory and national registries (Sweden and The Netherlands); (2) auditing strategies; and (3) length of follow-up. These variances might explain why the registry was associated with major morbidity. The current study tried to correct for these potential influences by testing the association of the four registries in the logistic regression analyses. Seventh, no data on adjuvant therapy and survival are collected in the four audits/registries, whereby it was not possible to assess the impact of complications on the receipt of adjuvant chemotherapy and overall survival.

Nevertheless, the major strength of this study is that this relates to the first multicenter study that compared the surgical outcome after DP-PVR versus standard DP, providing valuable evidence regarding the surgical risks of DP-PVR in a large international cohort. These new insights can guide surgeons in preoperative and intraoperative decision making. Future studies should focus on the comparison of DP-PVR with either extended PD-PVR or TP-PVR, hereby taking into account the extent of venous involvement and the type of PVR, and investigating the surgical outcome, including the short- and long-term venous patency as well as the long-term oncological outcome.

## Conclusion

Pancreatic adenocarcinoma located in the pancreatic body with portomesenteric venous involvement can be safely managed with DP-PVR, considering the elevated but limited risk for additional major morbidity (24% vs. 18%) compared with standard DP without additional risk for mortality (1.7% vs. 1.2%).

## Appendix 1. Major differences in registry designs

**Table:**

	North America	Germany	Sweden	The Netherlands
Design	Multicenter	Multicenter	Nationwide	Nationwide
Participation	Voluntary	Voluntary	Mandatory	Mandatory
Auditing	No auditing, but data entry by independent reviewers^a^	Yearly auditing	Auditing each third year	Auditing performed once (2017)
Portomesenteric venous resection	Only ISGPS types 2–4 are registered	ISGPS types 1–4 are registered	ISGPS types 1–4 are registered	ISGPS types 1–4 are registered

*ISGPS* International Study Group for Pancreatic Surgery^3^

^a^The surgical clinical reviewers undergo annual certification testing to assure interrater reliability. They are guided by published variable definitions, annual courses, quarterly webinars, and daily variable question support
